# Improved methods to capture the total societal benefits of zoonotic disease control: Demonstrating the cost-effectiveness of an integrated control programme for *Taenia solium*, soil transmitted helminths and classical swine fever in northern Lao PDR

**DOI:** 10.1371/journal.pntd.0006782

**Published:** 2018-09-19

**Authors:** Walter O. Okello, Anna L. Okello, Phouth Inthavong, Tassilo Tiemann, Ammaly Phengsivalouk, Brecht Devleesschauwer, Alexandra Shaw, John Allen

**Affiliations:** 1 Division of Infection and Pathway Medicine, University of Edinburgh, Edinburgh, United Kingdom; 2 Australian Animal Health Laboratory (AAHL) Regional Program, CSIRO, Geelong, VIC, Australia; 3 Department of Livestock and Fisheries, National Animal Health Laboratory (NAHL), Vientiane, Lao PDR; 4 Tropical Forages Program, Centro Internacional de Agricultura Tropical (CIAT in Asia), Vientiane, Lao PDR; 5 Department of Epidemiology and Public Health, Sciensano, Brussels, Belgium; 6 Department of Veterinary Public Health and Food Safety, Faculty of Veterinary Medicine, Ghent University, Merelbeke, Belgium; 7 A P Consultants, Andover, United Kingdom; Instituto de Investigaciones Biomedicas, UNAM /Instituto de Neurologia y Neurocirugía, MEXICO

## Abstract

**Background:**

Control and elimination of zoonotic diseases requires robust information about their effect on both human and livestock health in order to enable policy formulation and the allocation of resources. This study aimed to evaluate the cost-effectiveness of controlling *Taenia solium* taeniasis/cysticercosis in both humans and pigs, and soil-transmitted helminths (STH) in humans by integrating their control to on-going human and animal health control programmes in northern Lao People’s Democratic Republic.

**Method:**

A cross-sectional study was carried out in 49 households, focusing on the prevalence of *T*. *solium* taenias/cysticercosis and soil transmitted helminths before and after a twelve month intervention. The village data was collected using a semi-structured questionnaire through a door-to-door survey. The village data was then projected to the wider northern Lao PDR population using stochastic modelling and cost-effectiveness ratio (after aggregating the net cost to capture both human and animal health parameters) and GDP per capita as a threshold, to determine the cost-effectiveness of the integrated control of *T*. *solium* taeniasis/ cysticercosis and STH, assuming linear scaling out of the intervention. The zoonotic DALY (zDALY) approach was also used as an alternative method of estimating the cost-effectiveness ratio of controlling *T*. *solium* taeniasis/cysticercosis in humans and pigs.

**Findings:**

Using cost-effectiveness analysis after aggregating the net cost and control of *T*. *solium* taeniasis/cysticercosis alone as the base case, the study found that simultaneous control of *T*. *solium* taeniasis/cysticercosis in humans and pigs, STH in humans and Classical Swine Fever (CSF) in pigs was USD 14 per DALY averted and USD 234 per zDALY averted using zDALY method hence considered highly cost-effective whereas controlling *T*. *solium* taeniasis/cysticercosis without incorporating STH and CSF was the least cost-effective (USD 3,672 per DALY averted). Additionally, the cost-effectiveness of controlling *T*. *solium* taeniasis/cysticercosis in people and pigs using zDALY as an alternative method was USD 3,662 per zDALY averted which was quite close to our findings using the aggregate net cost method.

**Conclusion:**

The study showed that control of *T*. *solium* taeniasis/cysticercosis alone in humans and pigs is not cost-effective in northern Lao PDR whereas control of STH is. Consequently, integrating *T*. *solium* taeniasis/cysticercosis control with other cost-effective programmes such as STH and CSF markedly improved the cost-effectiveness of the intervention. This is especially important in low resource countries where control of zoonotic neglected tropical diseases could be integrated with the human and animal health sectors to optimize use of the limited resources.

**Trial registration:**

Australia New Zealand Clinical Trials Registry (ANZCTR) ACTRN12614001067662.

## Author summary

A study was conducted in northern Lao PDR to ascertain the cost-effectiveness of controlling *Taenia solium* (*T*. *solium* taeniasis/cysticercosis) using five approaches namely: i) *T*. *solium* taeniasis/cysticercosis alone in the human population (the base comparator), ii) *T*. *solium* taeniasis/cysticercosi*s* and soil transmitted helminths (STH) in the human population, iii) *T*. *solium* taeniasis/cysticercosi*s* alone in the human and pig population, iv) *T*. *solium* taeniasis/cysticercosis in the pig population and STH in humans, and v) *T*. *solium* taeniasis/cysticercosis, STH and Classical Swine Fever (CSF) in humans and pigs. Using cost-effectiveness ratio (after aggregating the net cost and using zDALY approach as an alternative method), the study found that the simultaneous control of *T*. *solium* taeniasis/cysticercosis, STH and CSF in human and pig population was USD 14 per DALY averted and USD 234 per zDALY averted thus considered highly cost-effective whereas control of *T*. *solium* taeniasis/cysticercosis alone in the human and pig population was the least cost-effective as it was found to be USD 3,672 per DALY averted using the aggregate net cost method and USD 3,662 using the zDALY approach,. We concluded that inclusion of STH and CSF to *T*. *solium* taeniasis/cysticercosis mitigation efforts drastically improved the overall cost-effectiveness of the intervention in northern Laos where all the three diseases are endemic.

## Introduction

*Taenia solium* taeniasis*-*cysticercosis complex is a zoonotic Neglected Tropical Disease (zNTD) found throughout many parts of Asia, Africa and Latin America, particularly where pigs and humans co-exist in areas of poor sanitation and hygiene [1–2]. Being the most important food-borne parasite worldwide and ranked fourth among all food-borne pathogens [3], there is a growing requirement for improved understanding of the global burden and demonstration that control is cost-effective [4].

The World Health Organization (WHO) has promoted a scale up of *T*. *solium* taeniasis/cysticercosis control and elimination efforts by 2020, buoyed by its status as one of six diseases identified as ‘potentially eradicable’ by the International Task Force for Disease Eradication [5]. Amongst other things, the task force recommends integrated control strategies, consideration of economic factors and assessment of the impact of mass drug treatment on co-endemic parasitic diseases such as soil-transmitted helminths (STH) to help promote support for eradication [5]. Following this, there is broad consensus that the economic analysis of zoonoses control programmes should be based on a holistic measurement of ‘total societal benefits’ as compared to simply calculating the total costs of controlling disease in humans and in animal reservoirs [6]. This requires an understanding of the level of integration [7–8] and comprehensive economics metrics to compare cost-effective control methods [9]. In the past, integrated control of neglected tropical diseases such as trachoma and primary healthcare [10], schistosomiasis and STH using common drugs [11], rabies in the animal health sector [12] among others have been attempted with varying results. Although, a policy of integrated control of neglected tropical diseases is highly beneficial [13–15], studies on the cost-effectiveness of such an approach are rare [16].

This study aims to quantify the overall cost-effectiveness of a successful ‘rapid impact’ *T*. *solium* taeniasis/cysticercosis control programme that treated both pigs and humans, resulting in a significant (p < 0.001) *T*. *solium* taeniasis/cyst*icercosis* reduction of 77.4% over a sixteen-month period in a smallholder farming system in Southeast Asia typically characterized by reliance on family labour, small farm size, minimal input and low income [17]. Apart from the *T*. *solium* taeniasis/cysticercosis control program in Laos, other studies have shown that the prevalence of epilepsy in Lao PDR is 7.7 per 1,000 people [18] with high fatality rates [19–20] and low levels of healthcare [21–22]. To date, whilst models have suggested that a combined therapeutic approach in both pig and human hosts will result in the greatest sustained impact on parasite levels [23], few research interventions have explored this concept in practice [24–25]. In this study we also evaluated combined human mass drug administration (MDA), oxfendazole deworming of pigs and vaccination of pigs using TSOL18 and Classical Swine Fever (CSF) vaccines based on the holistic One Health intervention undertaken by Okello et al. (2016) [17] which aimed to optimize its total societal value through integration into existing district-level programmes driven by the Lao PDR Ministries of Health and Agriculture in the target area and carrying out joint activities. On the human side, this was achieved through two rounds of community mass drug administration (MDA) with a three day albendazole 400mg protocol to decrease the level of environmental contamination with tapeworm eggs and incorporate STH control and thus align with the Ministry of Health’s ongoing STH control objectives [26–27]. During the MDA, local government medical staff visited all participating households for five consecutive days administering drugs, monitoring for adverse reactions and carrying out screening exercises for epilepsy. The human health intervention excluded pregnant women and children less than six years old. The standard porcine anti-cysticercosis intervention (which excluded pregnant or lactating pigs as well as those earmarked for sale), consisting of TSOL18 vaccination and oxfendazole (OFZ) at 30mg/kg [28–29], also incorporated Classical Swine Fever (CSF) vaccination, an important porcine production-limiting disease in Southeast Asia [30] and a priority disease for the Lao PDR Ministry of Agriculture. Classical Swine Fever, especially genotype 2.2, is endemic in northern Lao PDR and it is characterized by abortions and stillbirths of sows, as well as lack of appetite, anorexia, and high-mortality (can reach 100%) among nursery pigs [31–32]. It is hoped that this methodology and findings will help drive similar cost analyses for *T*. *solium* taeniasis/cysticercosis and other zNTD interventions, whilst simultaneously encouraging the consideration and inclusion of possible collateral benefits into control of other diseases under a true One Health approach. Consequently, to make this study have a wider applicability, a research question and null hypothesis were developed. The research question was ‘how does the inclusion of STH and CSF affect the cost-effectiveness of *T*. *solium* taeniasis/cysticercosis control?’, and based on this question, the null hypothesis was that inclusion of STH and CSF has no significant impact on the cost-effectiveness of *T*. *solium* taeniasis/cysticercosis control. The base case was the *T*. *solium* taeniasis/cysticercosis control alone without inclusion of STH and CSF while the comparators were the *T*. *solium* taeniasis/cysticercosis control strategies that included STH and CSF.

## Methodology

### Study area

The study was conducted in Mai district, Phongsaly province in the northern region of Lao PDR. The target area consisted of a homogenous Tai Dam population of around 400 people in 55 households, where the pre-intervention *T*. *solium* taeniasis/cysticercosis prevalence was found to be one of the highest recorded globally to date [33]. The Tai Dam are an ethnic group from northern Lao PDR, Vietnam, Thailand and China with strong cultural ties to animal sacrifice, using pigs, chickens and buffalo during various ceremonies and festivities that connect them with a higher spiritual world [34].

### Data collection

After seeking a written consent and conducting a door to door household census, a semi-structured questionnaire was used to determine household characteristics, pig productivity and human health parameters; including reporting on epilepsy through screening [35–36] in 49/55 (89.1%) of village households. The initial baseline survey, conducted in October 2014, included a 12 month recall for livestock productivity data regarding pig production. During the subsequent 18-month intervention [17], economic monitoring occurred via every six months updates on changes in the village pig population (births, deaths, sales, purchases etc), human health parameters, and response to both the human and pig interventions which were concurrently undertaken.

### Economic evaluation of the intervention costs and benefits

The total societal view, where all resources are captured irrespective of who incurred or benefited from them, was central to cost computation in this study. The intervention strategies being compared in this study were: i) *T*. *solium* taeniasis/cysticercosis alone in the human population (the base case), ii) *T*. *solium* taeniasis/cysticercosis and soil transmitted helminths (STH) in the human population, iii) *T*. *solium* taeniasis/cysticercosis alone in the human and pig population, iv) *T*. *solium* cysticercosis in the pig population and STH in humans, and v) *T*. *solium* taeniasis/cysticercosis, STH and Classical Swine Fever (CSF) in humans and pigs. These interventions represented all the possible scenarios public health policy makers would face in regards to control of *T*. *solium* taeniasis/cysticercosis in Laos; intervention strategies two to five were the comparators. The questionnaire captured both monetary and time expenditures borne by village inhabitants (private costs) resulting from symptoms or disease associated with *T*. *solium* taeniasis/cysticercosis or STHs (direct costs of health seeking treatment). The questionnaire also captured private costs incurred by smallholder farmers from pig rearing, via gross margin analysis of the pig enterprise in the target area. Public (project) costs were allocated to either the human or pig cost centres using a micro-costing approach [37], enabling their analysis as a constituent of the overall project cost without double counting. Capital depreciation, which was the only capital cost, was estimated using the straight line method [38] and aggregated amongst the cost centres. Examples of the human intervention project cost centre included the cost of albendazole tablets, capital depreciation and logistical costs. The project costs incurred from the pig intervention included the cost of oxfendazole, TSOL18 and CSF vaccine, plus other recurrent expenditures. Also, secondary data such as cost of treatment and drugs were fitted to gamma distribution using the fitdistrplus package for R [39] and analysed using a Monte Carlo simulation to estimate the 95% uncertainty interval. For the purposes of analysis, the costs and benefits were divided into human (non-monetary and monetary) and pig (monetary), although execution of both interventions was combined.

### Calculating the total human benefit of the intervention

DALYs represent the non-monetary human disease burden, calculated through combining the years of life lost due to premature death (YLL) and years lived with disability (YLD) [40–41]. The epidemiological parameters used for the DALY calculations of neurocysticercosis (NCC) and STH were obtained using a combination of empirical data derived from household questionnaires and secondary literature sources inputted into R software (version 3.2.2) [42]. Preference was given to secondary data obtained from the study area or in other districts of Laos. However data from south-east Asia was used in cases where there was no information available in the study area or other parts of Laos.

Since the accuracy of DALY estimates rely heavily on the information obtained for its computation, secondary data were fitted to uniform and beta distribution using the fitdistrplus package for R [43] and analysed using a Monte Carlo simulation, allowing for estimation of uncertainty to the DALY estimate [44]. Also, the discount rate and social weighting (K and r values in the YLL equation) were set at zero to allow for comparison with other studies and the burden of *T*. *solium* taeniasis/cysticercosis and STH averted was represented as DALY [0, 0, 0] [40].

A door-to-door survey [45] was undertaken to estimate the number of epilepsy cases in the target area, with the prevalence converted to incidence by dividing it by illness duration [46]. The proportion of epilepsy due to NCC was estimated using secondary data, given the study did not diagnostically confirm reported epilepsy cases. The estimated STH prevalence within the target area [27] was combined with STH prevalence data from other northern Lao PDR provinces [47–48], then converted to incidence levels [48–49]. Tables 1 and 2 provide a summary of all epidemiological parameters that were used to estimate the non-monetary burden of *T*. *solium* taeniasis/cysticercosis and STH in the broader northern Lao PDR population.

**Table 1:** Epidemiological parameters used for computation of the burden of NCC.

Parameter	Value	Distribution	Data source
Population	1,141, 785	Fixed	[50]
Prevalence of epilepsy	7.7 per 1,000	Fixed	[18]
Proportion of epilepsy due to NCC	0.07	Uniform (0.002–0.158)	[51–53]
Case fatality ratio for NCC (%)	0.11	Beta (0.02–0.22)	[18]
Age of onset for age group 0–4 years	2.0	Fixed	[54]
Age of onset for age group 5–14 years	9.9	Fixed	[54]
Age of onset for age group 15–44 years	26.9	Fixed	[54]
Age of onset for age group 45–59 years	51.9	Fixed	[54]
Age of onset for age group over 60 years	73.6	Fixed	[54]
Average duration for age group 0–4 years	1.4	Fixed	[55]
Average duration for age group 5–14 years	2.0	Fixed	[55]
Average duration for age group 15–44 years	3.6	Fixed	[55]
Average duration for age group 45–59 years	2.8	Fixed	[55]
Average duration for age group over 60 years	1.6	Fixed	[55]
Disability weights for epilepsy treated	0.076	Uniform (0.047–0.106)	[56]
Disability weights for epilepsy untreated	0.424	Uniform (0.279–0.572)	[56]

**Table 2:** Epidemiological parameters used for computation of the burden of STH.

Parameter	Value	Distribution	Data source
Prevalence of Hookworm	0.46	Beta (0.45–0.47)	[27, 47–48]
Prevalence of *Ascaris lumbricoides*	0.12	Beta (0.12–0.13)	[27, 47–48]
Prevalence of *Trichuris trichura*	0.4	Beta (0.39–0.42)	[27, 47]
Case fatality ratio for STH	0.0014–0.08	Beta (0.000–0.001)	[57]
Age of onset for age group 0–4 years	2	Fixed	[58]
Age of onset for age group 5–14 years	10	Fixed	[58]
Age of onset for age group 15–44 years	30	Fixed	[58]
Age of onset for age group 45–59 years	50	Fixed	[58]
Age of onset for age group over 60 years	70	Fixed	[58]
STH duration	1	Fixed	[58]
Disability weight *Ascaris Lumbricoides*	0.067	Uniform (0.0108–0.1245)	[57, 59]
Disability weight trichuriasis	0.067	Uniform (0.0108–0.1245)	[57, 59]
Disability weight hook worm	0.063	Uniform(0.0041–0.1245)	[57, 59]

### Calculating the total livestock benefit of the intervention

The animal arm of the zoonotic disease burden is represented in this case by pig livestock production losses, incorporating the costs of both livestock death and morbidity such as lowered fecundity, weight loss leading to a reduced sale price, or carcass condemnation due to the presence of cysts. Losses to the pig production enterprise were determined via a ‘livestock production’ section of the household questionnaire which evaluated the numbers of pigs bought (including the prices they were bought), sold (including prices they were sold), died (including reasons for the death) and born per household over the given time period. A second element considered the private (borne by livestock keepers) and public (project) animal health expenditure in terms of both time and money, expressed as a component of the variable costs. The gross margin (expressed as the net benefit) was then calculated to determine the change in household income pre and post intervention according to the standard formula: Gross margin = [livestock output]–[variable cost] [60], where livestock output is defined as = [(animals and produce ‘out’)–(animals and produce ‘in’)] plus change in herd value. The change in herd value is expressed as [closing valuation (the total number of pigs at the end of the year multiplied by their value)—opening valuation (the total number of pigs at the beginning of the year multiplied by their value)]. The value of the pig is a function of its weight which is correlated with its age and health status; a pig’s weight is influential in determining its selling or buying price. Typically younger pigs (piglets and weaners) cost less to buy and sell compared to older pigs (growers, sows and boars). The value of the pigs which was not captured by the questionnaire was obtained from key informant interviews (which composed of 12 farmers, 9 traders, 3 animal health technicians and 2 veterinarians) by determining the most probable, minimum and maximum selling of each pig type and then using beta-PERT distribution in r software (mc2d r package) to determine the mean selling price in a smooth parametric distribution [42]. Variable costs include the costs of pig rearing incurred by the farmer plus any expenses of project participation (for example repair of pig pens).

### Projection of findings to the larger northern Lao PDR region

The cost-benefit analysis was projected to the broader northern Lao population over the course of one year in order to estimate the cost-benefit of control at a regional level that would more accurately reflect future control programmes assuming linear scaling. The total human population in the four Northern provinces considered for this projection (Phongsaly, Huaphan, Luang Prabang and Oudomxay) was 1,141,785; comprising 572,211 females (0–4 years age group had 71,194 females, 5–14 years age group had 150,213 females, 15–44 years age group 261,812 females, 45–59 years age group had 54,544 females and over 60 years age group had 34,448 females) and 569,574 (0–4 years age group had 71,766 males, 5–14 years age group had 154,355 males, 15–44 years age group had 258,017 males, 45–59 years age group had 53,540 males and over 60 age group had 31,896 males) males [50].

The number of pig rearing households, total number of pigs in each province and the average number of pigs per household in each of the four provinces was obtained from the Lao agricultural census [61]. In the four northern Lao provinces there were a total of 104,700 pig rearing households and the total number of pigs was 351,200. Computation of the gross margin per household entailed adjusting the income from the pig enterprise depending on the mean number of pigs per province, since the gross margin is highly dependent on the herd size.

### Analysing the total societal benefit of the intervention in relation to its total cost

The overall capacity of a public health intervention to prevent unwanted human health outcomes (such as mortality and prolonged morbidity resulting from disease presence) may be indicated by the number of DALYs averted [62]. The total cost-effectiveness of this *T*. *solium* taeniasis/cysticercosis control programme, in relation to the total costs and benefits accrued in both the human and pig arms of the intervention, were evaluated using cost-effectiveness ratio a standard measure of cost utility analyses [63]. However, since the study had both costs from the agricultural and health sectors, the aggregated net cost was calculated by subtracting the reduced human health cost (i.e. decrease in expenditure incurred by medical services, patients and their households), reduced animal health losses (i.e. mortality, weight etc.), reduced animal health expenditure (i.e. decrease in expenditure incurred by veterinary services and farmers) from the project cost as shown in Eq 1 [61].

$$\mathrm{NC}/\mathrm{DALY}\;\mathrm{averted}=\left({\mathrm{CI}‑{\mathrm{RE}}_{H}‑\mathrm{RL}}_{A}\right){/\mathrm{RB}}_{H}$$ (1)

Where NC/DALY averted is the net cost per DALY averted for each intervention, CI is the total project cost (in USD), RE_H_ is the reduced human health expenditure (in USD), RL_A_ is the reduced animal health losses, and RB_H_ is the DALY averted. The zoonotic DALY (zDALY) was also used as an alternative approach to estimate the cost-effectiveness ratio of controlling *T*. *solium* taeniasis/cysticercosis [64–65]. Just like the aggregate net cost method, the zDALY also provides a framework for combining the human burden and the losses incurred by livestock keepers in single metric and it does this by considering the monetary impact on livestock keepers in terms of a time trade-off, in the sense of the value of people’s time required to recoup these losses, and by using gross domestic product (GDP) as a numéraire, it convert these into a non-monetary time unit called ‘animal loss equivalent’ (ALE) [65]. Equally, the reduced expenditure on health due to an intervention is converted into ‘health loss equivalent’ (HLE). Another method for analysing total societal benefit of controlling zoonotic diseases is the separable cost method [16]. Although the main method used in this study was aggregated net cost, zDALY approach was also used to; i) capture the cost of zDALY averted for *T*. *solium* taeniasis/cysticercosis, and ii) costs per zDALY averted across all diseases rather than just for *T solium* utilising the ALE component of CSF.

To estimate the cost-effectiveness of controlling *T*. *solium* taeniasis/cysticercosis, the study applied the WHO cost-effectiveness thresholds, which considers an intervention as ‘highly cost-effective’ if the cost per DALY averted is less than the country’s GDP per capita; ‘cost-effective’ if the cost per DALY is between one and three times the GDP per capita; and ‘not cost-effective’ if the cost per DALY exceed three times the country’s GDP per capita [66]. In Lao PDR the GDP per capita at the time of the study was USD 1,793 [67]. Nominal current (year 2015 without adjusting for price inflation) market prices were used throughout for both disease control costs and livestock production costs and returns, thus reflecting the realities facing the health and veterinary services, pig producers and human patients in Lao. Accordingly the GDP value selected was also the nominal, or Atlas value, rather than being in international dollars adjusted for purchasing power parity.

### Ethics statement

Ethical approval for this study was granted by the Lao PDR Council of Medical Science National Ethics Committee for Health Research (NECHR), approval number 013/NECHR, and Australia’s Commonwealth Scientific and Industrial Research Organisation (CSIRO) Animal, Food and Health Sciences Human Research Ethics Committee (CAFHS HREC), approval number 13/10. The study was registered with the Australia New Zealand Clinical Trials Registry (ANZCTR), trial number ACTRN12614001067662.

## Results

### Descriptive socioeconomic characteristics

From a total of 55 target area households, 49 (89%) were included in the study; six households had relocated during the course of the study, hence were not included in the final calculations. The total number of people in the 49 households was 375 comprising 178 males (0–4 years age group had 20 males, 5–14 years age group had 45 males, 15–44 years age group 85 males, 45–59 years age group had 18 males and over 60 years age group had 10 males) and 197 females (0–4 years age group had 27 females, 5–14 years age group had 55 females, 15–44 years age group had 86 females, 45–59 years age group had 18 females and over 60 age group had 11 females). According to information obtained from key informant interviews, all pigs were left to roam freely, with the larger ones penned during the rice harvesting season. Questionnaires indicated the average weight of sold pigs was around 23 kilograms (kg) and the price of pork around USD 3/kilogram. Out of the 49 households, 41 (83%) did not have toilets, whereas 8 (16%) had toilets and used them.

**Fig 1:**
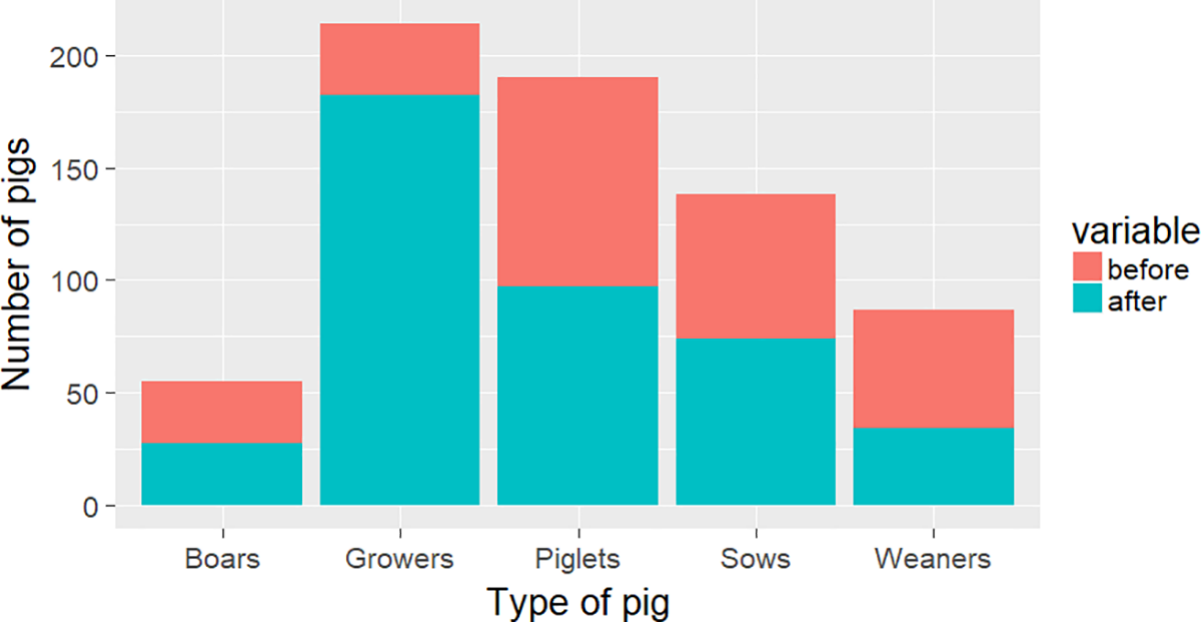
Pre and post intervention pig numbers in the target area.

The baseline pig population in the target area was 270, with a mean number of 5.2 (standard deviation 4.9) pigs per household. The herd structure consisted of 28 boars (10%), 64 sows (24%), 53 weaners (20%), 32 growers (12%) and 93 (34%) piglets. Post intervention, pig numbers had increased by 53% to 414, with a mean number of 8.4 (SD 6.1) pigs per household. Changes in the herd structure, particularly in the grower category, were evident with 27 boars (7%), 74 sows (18%), 34 weaners (8%), 182 growers (44%) and 97 piglets (23%) post intervention (Fig 1). Before intervention, mortality was 48.3% (185/386) comprising of 138 piglets, 32 weaners, 7 growers, 5 sows and 3 boars. After the intervention the mortality dropped to 8.4% (37/440) comprising of 16 piglets, 9 weaners, 6 growers, 3 sows and 3 boars. There were 386 responses on the reasons for death and these were mentioned as follows; diseases (69%), lack of feed (13%), accidents (1%), dog bites (2%), poisoning (1%), gunshot wounds (1%), and piglets dying from low milk supply from sow during lactation (1%) and still birth (12%).

### Project costs for the combined human and pig intervention

#### Costs (capital and recurring) incurred by the project

During the first two rounds of control, in October 2014 and March 2015, the human and pig interventions were undertaken simultaneously. This resulted in combined project expenditures for these activities, with the final October 2015 pig intervention treated as a single activity. The only capital cost was for vehicle depreciation, while the recurrent costs included drugs, vaccines, fuel and staff costs. The total capital and recurrent expenditure for the human cost centre was USD 3,498, and the pig cost centre USD 21,085. The pig cost centre was further divided into TSOL18, OFZ and CSF vaccination.

#### Direct financial costs incurred by the project

The human arm of the intervention consisted of two rounds of MDA with an albendazole 400 mg triple dose protocol and one monitoring exercise, as described by Ash et al (2015) [27]. The direct financial project costs for these activities were the cost of albendazole tablets (1,048 tablets at USD 0.1 each) delivered to 375 human participants. The pig intervention (TSOL18 plus OFZ plus CSF) was administered four times over the intervention period (three vaccination activities and one monitoring exercise), with direct financial costs including the cost of CSF vaccine and OFZ. The CSF vaccine was bought in bulk from the government veterinary laboratory based in Vientiane, at a total cost of USD 100 (833 doses at USD 1.2 per 10 doses), with a total number of 828 pigs vaccinated during one year of the intervention. Although in this case the TSOL18 vaccine was donated (MWL Lightowlers, University of Melbourne), for the purposes of this analysis the cost has been included as the expected wholesale commercial cost of around 0.50 USD/dose, 95% uncertainty interval (UI: USD 0.4–0.6) (personal communication Galvmed 2017). The cost of the various component interventions have been summarised in Table 3.

**Table 3:** Total public (project) costs of the human-pig intervention.

Cost item	Human intervention (USD)	Pig intervention (USD)			Total cost (USD)
	Mass drug administration (MDA)	TSOL18 vaccination	Deworming with oxfendazole	Classical Swine Fever vaccination	
Capital expenditures					
Vehicle depreciation	250	250	250	250	1,000
Recurrent expenditures					
Value of field staff participation human MDA	1,378	0	0	0	1,378 (95% UI: 1,239–1,599)
Value of field staff participation during pig intervention	0	5,052	5,052	5,052	15,156 (95% UI: 12,671–17,791)
Value of field staff participation during monitoring	148	148	148	148	592 (95% UI: 525–667)
Local facilitation fees	75	75	75	75	300 (95% UI: 186–442)
Total cost of human drugs	105	0	0	0	105
Cost of Classical Swine Fever vaccine	0	0	0	100	100
Cost of TSOL18 vaccination	0	810	0	0	810 (95% UI: 727–929)
Cost of oxfendazole dewormer for pigs	0	0	366	0	366
Additional vehicle hire	315	315	315	315	1,260 (95% UI: 1,184–1,391)
Total fuel used	500	500	500	500	2,000 (95% UI: 1,590–2,619)
Questionnaire development, pre-testing and printing	38	38	38	38	152 (95% UI: 107–206)
Laboratory diagnostics	464	0	0	0	464 (95% UI: 299–939)
Miscellaneous	225	225	225	225	900 (95% UI: 818–1,021)
Total amount in USD	3,498	7,413	6,969	6,703	24,583

### Human health results

#### Non-monetary cost of NCC and STH (DALYs)

The clinical manifestations of epilepsy are well known in northern Lao PDR, named ‘*bah moo’* (crazy pig) in the local language based on the likeness of sufferers to salivating pigs. Door-to-door household surveys in the target area revealed approximately 0.8% of inhabitants self-reported suffering from epilepsy (3/375), based on a clinical description of seizures with or without mouth foaming. Post intervention epilepsy prevalence was reported at 0.2% (1/375); equivalent to 2 people per 1,000. Projecting these values to the broader northern Lao PDR region, the number of people estimated to be suffering from epilepsy pre-intervention was 9,134, resulting in an estimated 2,404 patients with estimated NCC-induced epilepsy in the broader northern region.

Entering these parameters into R software [40], the pre-intervention DALY from NCC was estimated to be 4,958 (95% UI: 4,940–4,975), with a total DALYs of 434 per 100,000 person years. It was assumed that there would be 2,284 people suffering from epilepsy post-intervention; hence the estimated number of people that would suffer from NCC-induced epilepsy in northern Lao PDR was 580. The total DALYs due to NCC was 1,480 (95% UI: 1,475–1,485) post-intervention; and the total DALYs per 100,000 person years due to NCC-induced epilepsy was 130 post-intervention in northern Lao PDR, representing a 70% reduction in the disease burden. The epidemiological parameters of the STH were computed in R (v 3.1.1) after coding, yielding the DALY figures pre- and post-intervention for each of the three diseases. Consequently the total DALYs averted was estimated to be 59,556 in northern Lao PDR, representing an overall reduction of 63% of the total disease burden due to NCC and STH as shown in Table 4.

**Table 4:** DALY averted for NCC and STH.

Disease	DALY Pre-intervention	DALY Post-intervention	DALY averted	Percentage reduction in DALY burden
Trichuriasis	35,250	17,923	17,327	49
Hookworm	37,786	11,168	26,618	70
Ascariasis	17,237	5,104	12,133	70
NCC	4,958	1,480	3,478	70
Total	95,231	35,675	59,556	63

### Patient cost of NCC and STH

#### Cost of NCC-associated epilepsy treatment

Data obtained from the door-to-door survey revealed that only 33% identified persons with epilepsy (PWE) sought healthcare (traditional healer), spending an average of USD 17 (95% UI: 14–19) on treatment per year. Sixty seven percent of PWE did not seek any form of treatment. Based on this, an estimated 793 (33%) NCC-associated epileptic patients sought treatment, at a cost of USD 13,481/year, before the intervention. Post intervention, the number of NCC patients in northern Lao PDR seeking healthcare was estimated to be 191 at a cost of USD 3,247/year, resulting in a post-intervention difference of USD 10,234 in healthcare expenditure as a result of NCC-associated epilepsy.

#### Cost of STH treatment

According to the data obtained from the household surveys using one year recall, 56% (210/375) participants’ self-reported symptomatic diarrhoea and anaemia, with 19% of these (40/210) reporting to have sought health care. Of the individuals that sought healthcare, 6 went to the medical doctor, spending an average of USD 0.6 (UI: 0.4–0.8) each, and the remaining 34 patients went to the village soothsayer, spending an average of USD 0.1 (95%UI: 0.03–0.19) each. Using a gamma distribution, the mean expenditure incurred when seeking healthcare (both from the medical doctor and soothsayer) was USD 0.3 (95% UI: 0.1–0.5). Data from Ash et al. (2015) [27] revealed that the mean STH prevalence in the target area was 53%, consequently an estimated 605,146 individuals were affected by STH, with a total health expenditure of USD 34,493, across the four northern Lao PDR provinces pre-intervention. The estimated post-intervention STH prevalence in the target area was 9.9% [43], projecting to an estimated 113,037 people across the broader region, with a treatment expenditure of USD 6,443. Based on the difference in the amount of money spent on treating NCC and STH within the informal private health sector, the difference in health expenditure after the intervention was USD 28,050. Consequently, the total amount of health expenditure averted was USD 38,284.

### Pig intervention results

#### Pigs and pork products ‘out’

The value of pigs and pork products out constituted the total value in USD of all pigs sold, slaughtered or those given as gifts among the inhabitants of the village throughout the intervention. Ninety-six pigs (18 piglets, 10 weaners, 60 growers, 3 sows and 5 boars) were sold at a total value of USD 3,519 in the 12 months leading up to the intervention. Post intervention, this figure had risen to 102 pigs (10 piglets, 5 weaners, 80 growers, 2 sows and 15) sold at total value of USD 8,364. Baseline slaughter numbers (i.e. slaughtered and consumed without condemning) were 21 at a total value of USD 2,232, which at post intervention had risen to 46 at a total value of USD 3,856. Other pigs transferred out of the village herd (gifts for other reasons) totalled 13, at a total value of USD 768 pre-intervention and largely consisted of younger pigs, increasing to 19 pigs and a value of USD 1,239 post intervention. Therefore total value of pigs sold, slaughtered or gifted out before and after the intervention was USD 6,519 and USD 13,459 respectively; a difference of USD 6,940.

#### Pigs and pork products ‘in’

The value of pigs and pork products into the target herd consisted of the sum total value of all pigs purchased or that came into the herd for other reasons, for example received as gifts. In the 12 months leading up to the intervention, a total of 17 pigs (8 sows, 3 weaners and 6 growers) were purchased by the village households, at a total expenditure of USD 960. Post intervention analysis demonstrated that this figure had dropped by the end of the intervention, with 9 pigs purchased (5 sows, 1 weaner, 2 growers and 1 piglet) at a cost of USD 630. Eleven pigs (2 boars, 3 sows, 3 weaners, 1 grower and 2 piglets) were received as gifts pre-intervention, valued at USD 737, decreasing to gifts of 3 pigs (2 sows and 1 grower) worth USD 231 post intervention. Consequently, the overall total decrease in the value of pigs bought or gifted in the village over the intervention period was USD 837.

#### Gross margin from pig enterprise before and after the intervention

To complete the livestock output calculation, the change in herd value was calculated. This is difference between the closing valuation (total number of pigs at the end of the year multiplied by their value) and the opening valuation (total number of pigs at the beginning of the year multiplied by their value). Using one year recall, the total number of pigs one year before the baseline study (October 2013 to October 2014) was 256 (86 piglets, 50 weaners, 33 growers, 62 sows and 26 boars) valued at USD 12,471 (which represents the opening valuation). During the baseline year there were 270 pigs as mentioned earlier valued at USD 14,372 hence the herd value before the intervention was USD 1,901. After the intervention (closing valuation which was October 2015) there were 440 pigs valued at USD 19,395 and by subtracting the opening valuation (value of pigs at the start of the year which was October 2014 in this case) from the closing valuation the herd value post-intervention was USD 5, 024. To obtain the gross margin from the pig enterprise, the variable costs were deducted from the livestock output. These consisted of pen repairs, veterinary drugs and feedstuff. Before the intervention, the total variable cost was USD 67, decreasing to USD 27 after the intervention so that the gross margin before the intervention was USD 6,697, rising to USD 17,556 after the intervention (a difference of USD 10,859). This is equivalent to USD 137 and USD 358 per household from the pig enterprise before and after the intervention respectively, so that the average gain was USD 221, increasing the household gross margin from pig rearing by a factor of 2.6. Table 5 provides a summary of the gross margin results. Apart from the determination of the gross margin from the pig enterprise, the study found that the total loss from pig condemnation by owners at the time of slaughter before intervention was USD 233, decreasing to USD 6 post intervention. Consequently, the total benefit across all the households from pig production was USD 11,086.

**Table 5:** Village pig gross margin analysis.

Item	Total income (USD)	
	Pre-intervention	Post intervention
1. Pigs and pork products ‘out’		
Total value of sold pigs	3,519	8,364
Total value of slaughtered pigs	2,232	3,856
Total value of pigs gifted/stolen/disappeared	768	1,239
Subtotal: Value of pigs/pork products ‘out’	6,519	13,459
2. Pigs and pork products ‘in’		
Total value of purchased pigs	960	630
Total value of pigs received as gifts	737	231
Subtotal: Value of pigs/pork products ‘in’	1,697	860
3. Change in herd value		
Closing valuation	14,372	19,395
Opening valuation	12,471	14,372
Overall change in herd value	1,901	5,024
Livestock output	6,723	17,623
4. Variable cost to farmers	26	67
5. Total gross margin	6,697	17,556
6. Average HH income from pig enterprise	137	358

The gross margin from the pig enterprise was projected to the broader northern Lao PDR region by multiplying the equivalent total gross margin per household by the total number of pig-owning households in each province. The total gross margin for each province was then multiplied by 2.6 to estimate the total post intervention gross margin; resulting in a total gross margin of USD 9,398,720 before and USD 24,432 580 after the intervention as shown in Table 6; a projected difference of USD 15,033,860 at the regional level.

**Table 6:** Annual pig gross margin analysis in northern Lao PDR.

Province	Number of pig rearing households	Total gross margin pre-intervention across all households (USD)	Total gross margin post-intervention across all households (USD)
Huaphan	28,900	2,586,550	6,725,030
Luang Prabang	26,900	2,975,140	7,733,750
Oudomxay	26,100	1,855,710	4,823,280
Phongsaly	22,800	1,981,320	5,150,520
Total	104, 700	9,423,600	24,432,580

According to the village data, the annual post-intervention off-take was 24%, with 1.9% (1/51) slaughtered pigs condemned at home by their owners as the pork was grossly full of cysts. Projection of the same within four provinces in northern Lao revealed that of a total number of pigs slaughtered (84,488 or 1,934,775.2 kg of pork annually), an estimated 19,348 kg would be condemned, with a value of USD 58,043.

Consequently, the difference in the value of condemned pork before and after the intervention was estimated to be USD 23,990. Overall, the total benefit of controlling *T*. *solium* cysticercosis to the agricultural sector was USD 15,115,893, computed as the benefit of reduced pork condemnation (USD 23,990) plus increased regional household income (USD 15,057,850). It should be noted that the value of condemned pork was not part of the computation of the household income to avoid double counting of benefits.

### Extrapolated project cost

By estimating the human MDA coverage to be 63% [27] of 622,960 eligible participants over 4 years old, the total annual cost of MDA across all 4 Northern provinces was estimated to be USD 5,606,640. In the agricultural sector, the regional cost of treating pigs using TSOL18, CSF and OFZ was estimated to be USD 3,476,213, USD 3,125,013 and USD 3,722,053 respectively; totalling USD 10,323,279. Combining these figures resulted in a total project cost of USD 15,929,919 for the simultaneous control of *T*. *solium* taeniasis/cysticercosis, STH and CSF in the broader northern Lao PDR region.

### Determining the total cost-effectiveness of the intervention

By subtracting the livestock benefits (increased gross margin from pig enterprise and decreased value of condemned pork) and the averted cost in health expenditure from the project cost, the total cost from TSOL18, OFZ, CSF and human MDA was USD 833,785. Subsequently, the net cost-effectiveness of simultaneously controlling *T*. *solium* taeniasis/cysticercosis, CSF and STH was USD 14 per DALY averted. By computing the cost-effectiveness of *T*. *solium* taeniasis/cysticercosis control in the human population without integrating STH, and *T*. *solium* taeniasis/cysticercosis and STH (without addressing the pig population), the net cost-effectiveness of these approaches would be USD 1,609 and USD 93 per DALY averted respectively. By incorporating the pig intervention (TSOL18 and OFZ) with *T*. *solium* taeniasis/cysticercosis control only, the net cost-effectiveness was projected to be USD 3,672; by using the same regime but including STH in the calculations, the net DALY averted is USD 214. Table 7 provides a summary of the net cost per DALY averted for each of the *T*. *solium* taeniasis/cysticercosis control approaches.

**Table 7:** Net cost-effectiveness of the various intervention approaches.

Item	Intervention approach				
	Human intervention		Human and pig intervention		
	Control of *T*. *solium* only (A)	Control of *T*. *solium* + STH (B)	TSOL18, OFZ and (A)	TSOL18, OFZ and (B)	TSOL18, OFZ, CSF, (A) and (B)
a) Project cost (USD)	5,606,640	5,606,640	12,804,906	12,804,906	15,929,919
b) Livestock benefits (agricultural sector) (USD)	-	-	23,990	23,990	15,057,850
c) Health expenditure averted (health sector) (USD)	10,234	38,284	10,234	38,284	38,284
d) Sub-total (**a** *minus* **b** *minus* **c**) (USD)	5,596,406	5,568,356	12,770,682	12,742,632	833,785
e) DALYs averted (health sector)	3,478	59,556	3,478	59,556	59,556
f) Net cost per DALY averted (**d/e**) (USD/DALY)	1,609	93	3,672	214	14

By comparing the cost per DALY averted for each intervention approach with the Lao PDR GDP per capita as a measure of cost-effectiveness, it was found that the highly cost-effective approaches were i) control of *T*. *solium* taeniasis/cysticercosis in the human and pig populations, incorporating both CSF and STH control (14 USD/DALY averted) ii) control of *T*. *solium* taeniasis/cysticercosis and STH in the human population only (214 USD/DALY averted) and iii) control of *T*. *solium* taeniasis/cysticercosis in both the human and pig populations, incorporating STH control (93 USD/DALY averted). Control of *T*. *solium* taeniasis/cysticercosi*s* only (1,609 USD/DALY averted) was found to be cost-effective while the least cost-effective approach was incorporating the pig intervention (TSOL18 and oxfendazole) with *T*. *solium* cysticercosis control only (3,672 USD/DALY averted). Using zDALY approach, ALE for controlling *T*. *solium* taeniasis/cysticercosis was 13 and the HLE was 6. By adding DALY averted, which in this case was 3, 478 as estimated in Table 7, ALE and HLE, the *T*. *solium* taeniasis/cysticercosis zDALY was 3, 497. To compute the cost-effectiveness ratio of controlling *T*. *solium* taeniasis/cysticercosis in people and pigs without incorporating STH and CSF, the project cost (USD 5,606, 640 as estimated in Table 7) was divided by the zDALY yielding USD 3,662 per zDALY averted. Also, to compute the overall cost effectiveness ratio of combined control of all the three diseases (*T*. *solium* taeniasis/cysticercosis, STH and CSF), the ALE, HLE and DALY averted were totaled to determine the zDALY. Livestock benefit in terms of ALE was found to be 8,398, the HLE was 21 and the DALY averted was 59,556, thus the zDALY was 67,975 and the cost effectiveness ratio for the combined control of all the three diseases was USD 234 per zDALY averted; representing 13% of Laos per capita GDP and this was well within the WHO’s threshold of very cost-effective interventions.

### Sensitivity analysis

**Fig 2:**
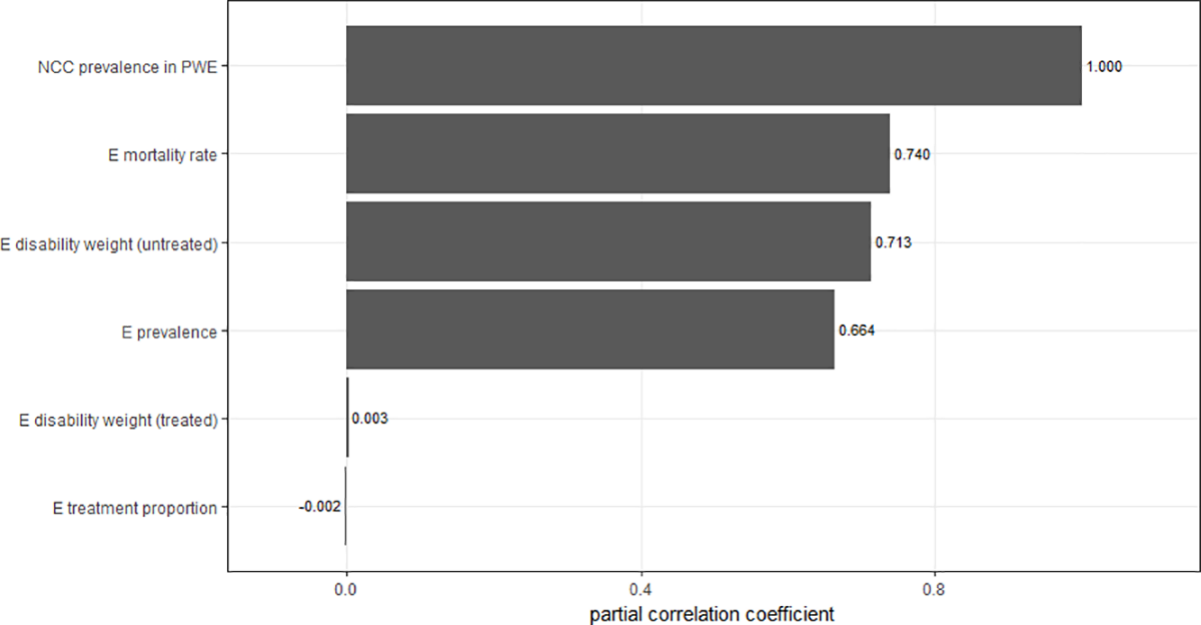
Sensitivity analysis of the neurocysticercosis associated DALY estimate for Lao. Epilepsy is denoted as E and persons with epilepsy as PWE in the diagram.

Sensitivity analysis using partial correlation coefficients showed that prevalence, mortality rate and disability weights were very influential in the disease burden models. For example, the prevalence of NCC, mortality rate, disability weight of the untreated, prevalence rate of epilepsy and disability weight of the epilepsy cases treated were the most influential in modelling the burden of *T*. *solium* taeniasis/cysticercosis as shown in Fig 2.

## Discussion

Although methods to estimate the costs of zoonotic disease to both livestock productivity and humans have been proposed [68–69], there has been no totally satisfactory conceptual framework for analyzing the total societal burden of zoonotic disease; that is the combined costs of disease from both the humans and animals [70]. The recently developed concept of the zDALY addresses this gap [65]. This study had both zoonotic (*T*. *solium* cysticercosis) and non-zoonotic (STH and CSF) diseases. *T*. *solium* cysticercosis as a zoonotic disease had YLL, YLD, HLE and ALE components of burden of disease, while STH as a non-zoonotic disease had YLL, YLD and HLE components. Given CSF is an animal disease it only had the ALE metric. Therefore based on the YLL, YLD, HLE and ALE disease burden metrics both aggregate net cost and zDALY approaches were used to estimate the changes in cost effectiveness ratio when externalities generated by treatment of *T*. *solium* taeniasis/cysticercosis are included. The aggregate net method revealed that the ‘highly cost-effective’ approach for northern region of Lao PDR is the combined human-animal approach incorporating *T*. *solium* taeniasis/cysticercosis control with two additional diseases; STH and CSF control (USD 14 per DALY averted) and zDALY approach corroborated this finding given the overall cost effectiveness ration of controlling all the three diseases (*T*. *solium* taeniasis/cysticercosis, STH and CSF) was USD 234 per zDALY averted representing 13% of the GDP per capita falling well within WHO’s threshold of very cost-effective interventions. Other cost-effective approaches included the human MDA intervention targeting both *T*. *solium* taeniasis/cysticercosis and STH (USD 93 per DALY averted), and the combined human-pig intervention that targeted both *T*. *solium* taeniasis/cysticercosis and STH (USD 214 per DALY averted). The least cost-effective intervention approaches were those that addressed *T*. *solium* taeniasis/cysticercosi*s* in isolation, regardless of whether this was in the human population (USD 1,609 per DALY averted), or jointly with an intervention in the pigs (USD 3,672 per DALY averted). Consequently, the results show that control of *T*. *solium* taeniasis/cysticercosis alone in humans and pigs is not cost-effective whereas control of STH in humans is. Also, the results obtained from using zDALY approach confirmed that it is not cost-effective to control *T*. *solium* taeniasis/cysticercosis alone in humans and pigs without incorporating STH and CSF in northern Lao; zDALY metric was very close to the findings from the aggregate net cost method. Also, the null hypothesis was rejected given that addition of STH and CSF markedly improved the overall cost effectiveness of controlling *T*. *solium* taeniasis/cysticercosis. Therefore, this study concluded that integrating *T*. *solium* taeniasis/cysticercosis control with other cost-effective programmes is recommended to effectively control it in Laos.

Information obtained from the semi-structured questionnaire supported previous findings that revealed smallholder pig rearing to be an important farming activity in the study area as also revealed by Bardosh et al (2014) [71]. The intervention resulted in improved pig productivity, seen as an increase in the average number of pigs reared per household from 5.2 to 8.4 after 12 months of the intervention, and a reduction in pre-weaning mortality from 48.3% to 8.4% due to CSF vaccination; low mortality was probably the main course of increased number of growers as most piglets were surviving and reaching this age. Apart from CSF vaccination, deworming of pigs (especially free ranging ones) with OFZ potentially played a role in improving the overall cost effectiveness of the intervention by protecting pigs from new *T*. *solium* cysticercosis infections thus protecting humans; as well as improving the health of pigs as it has an effect on nematode infections which are a source of disease and production losses. These results corresponded to the increased gross margin from the pig enterprise; remarkably the greatest production age increase was seen in growers, from 12% to 44% of the overall herd composition, highlighting the importance of integrating disease interventions into future pig productivity improvement projects. In the study area, the combination of animal health interventions with the availability of improved feeding, which had been established prior to the intervention, allowed farmers to take full advantage of the production capacity of their livestock, once animal health had been restored. Although the human health benefits alone fully justify the investment as demonstrated through the economic impact of averted DALYs, the combination of such an intervention with improved production approaches adds considerable value to the overall intervention. It might also give an additional incentive to farmers if the effect is large enough for them to notice the production–and in consequence economic–benefit. Also, vaccinating pigs with TSOL18 ensured a lifetime immunity to *T*. *solium* cysticercosis for pigs, reducing the risk of acquiring infection long term.

This study has shown that the inclusion of approaches that are effective against pig production diseases such as CSF has played a major role in increasing the cost-effectiveness in regions where *T*. *solium* cysticercosis and CSF are co-endemic. To achieve high cost-effectiveness in future, pig vaccination against *T*. *solium* cysticercosis could be done together with CSF or an equivalent bivalent ‘One Health’ vaccine developed for regions where CSF is endemic; *T*. *solium* cysticercosis does not typically affect pig productivity, it will be difficult to convince farmers to pay for *T*. *solium* cysticercosis vaccine unless they are likely to be penalized for cystic pork. Equally, where meat inspection practices are not well managed, *T*. *solium* cysticercosis interventions should focus on diseases or management practices that decrease pig mortality (pre-weaning mortality in particular), so as to achieve a higher survival rate and thus increase the overall livestock productivity benefits of the *T*. *solium* cysticercosis intervention.

There is a need for sharing resources between agricultural and health sectors, especially where the inclusion of secondary diseases such as STH and CSF play a major role in the benefits accrued to each sector. Joint disease control is a critical component of enhancing household health, wealth and overall wellbeing, given the biggest beneficiaries are the affected households. Unfortunately, integrated sectoral approaches under the One Health movement are rare, despite an acknowledged need to tackle societal problems such as zNZDs in a comprehensive manner [72]. A major reason observed for the lack of sustainable One Health approaches in veterinary public health is related to the concept of who should fund what, particularly where cost sharing between sectors is expected. However, this study clearly demonstrates that integrated actions at a larger scale are significantly more cost effective than ‘vertical’ disease approaches that address issues individually, and thus should be the guiding principle for addressing future *T*. *solium* taeniasis/cysticercosis interventions, or those against the zNTDs more generally.

This study has limitations, the primary observation being the large amount of secondary data used to simulate and estimate the cost-effectiveness of controlling *T*. *solium* taeniasis/cysticercosis, STH and CSF in the northern Lao region after assuming a linear scaling out of the intervention. The use of significant secondary data sources is not without precedent for estimations of *T*. *solium* taeniasis/cysticercosis burden [73–74], and highlights the current dearth of data globally for this disease resulting from and contributing to its neglected status. Further studies are needed to establish the *T*. *solium* taeniasis/cysticercosis, STH and CSF prevalent regions in northern Lao PDR, or indeed the broader southeast Asia region more generally; it would be prudent to focus initially on potential hyper-endemic *T*. *solium* taeniasis/cysticercosis ‘hotspots’, identified by a combination approach of both social and epidemiological methods. A second limitation, when looking at the aggregated societal benefits and the net monetary benefit, high livestock benefits may mean that monetary benefits exceed monetary costs. This would show that the programme is dominant: effective and cost-saving; in this situation, calculating incremental cost-effectiveness ratio is not relevant. This is a difficult result to interpret, or rank, and could have the unwanted effect of skewing the allocation of cost entirely towards the livestock sector, since livestock benefits outweigh costs. This would be a particularly unhelpful outcome, as the strength of this intervention is that it simultaneously deals with both the human and livestock disease reservoirs, resulting in greater sustainability. However other methodologies such as zDALY can be used to estimate monetary losses in livestock which can then be incorporated into the DALY estimate particularly if the intervention only involves zoonotic diseases. Other limitations include use of the village data with small sample size, use of data from a hyper-endemic foci to project the cost effectiveness of the intervention and lack of definitive diagnosis of NCC. Consequently the attribution of NCC to epilepsy in northern Lao PDR might be lower than stated in this study and further studies are needed to find out if this is the case and whether more *T*. *solium* taeniasis/cysticercosis hyper-endemic foci exist in northern Lao PDR. However, information obtained from the study area coupled with the sensitivity analysis on the assumptions used to estimate the DALY provides a good basis of understanding the impact of simultaneously controlling *T*. *solium* taeniasis/cysticercosis, STH and CSF.

## Conclusion

Control of *T*. *solium* taeniasis/cysticercosis in the northern Lao PDR is currently heavily dependent on therapeutic interventions in the human or pig populations–ideally both–to reduce the disease prevalence. However, sustainable control of *T*. *solium* taeniasis/cysticercosis should not be taken in isolation; incorporating the control of other pig production diseases (such as CSF) and/or soil transmitted helminth control is recommended to maximize the intervention cost-effectiveness. This is especially true for interventions that the farmer is expected to pay for; incorporating production-impacting diseases into *T*. *solium* taeniasis/cysticercosis control will incentivize farmers to pay for its control. This cost-effectiveness analysis clearly shows that controlling *T*. *solium* taeniasis/cysticercosis in isolation is not cost effective, and more holistic, innovative methods to build zNTD control into existing human health or livestock production and development programmes would be beneficial.

## Supporting information

S1 Checklist STROBE checklist. (DOC)

S2 Checklist CHEERS checklist. (DOC)
