# Supplementary material for: Improved methods to capture the total societal benefits of zoonotic disease control: Demonstrating the cost-effectiveness of an integrated control programme for Taenia solium, soil transmitted helminths and classical swine fever in northern Lao PDR
Source: PLoS Negl Trop Dis. 2018 Sep 19;12(9):e0006782. doi: 10.1371/journal.pntd.0006782 (PMC6185856; doi:10.1371/journal.pntd.0006782)
Supplement: S1 Checklist — (DOC) [file pntd.0006782.s001.doc]

STROBE Statement—Checklist of items that should be included in reports of ***cross-sectional studies***

|  | Item No | Recommendation |
| --- | --- | --- |
| **Title and abstract** | 1 | (*✓*) Indicate the study’s design with a commonly used term in the title or the abstract |
|  |  | (*✓*) Provide in the abstract an informative and balanced summary of what was done and what was found |
| Introduction | | |
| Background/rationale | 2 | (*✓*) Introduction, paragraph 1and 2. |
| Objectives | 3 | (*✓*) Introduction, paragraph 3. |
| Methods | | |
| Study design | 4 | (*✓*) Methods, subtitle “Data collection”, paragraph 1.  (*✓*) Methods, subtitle “Study area”, paragraph 1.  (*✓*) Methods, subtitle “Data collection”, paragraph 1.  (*✓*) Methods, subtitle “Economic evaluation of intervention costs and benefits”, paragraph 1.  (*✓*) Methods, subtitle: “Calculating the total benefit of the intervention”, paragraph 2. |
| Setting | 5 |  |
| Participants | 6 |  |
| Variables | 7 |  |
| Data sources/ measurement | 8 |  |
| Bias | 9 | (*✓*) Methods, subtitle “Data collection”, paragraph 1. |
| Study size | 10 | (*✓*) Methods, subtitle “Data collection”, paragraph 1. |
| Quantitative variables | 11 | (*✓*) Methods, subtitle “Economic evaluation of intervention costs and benefits”, paragraph1.  (*✓*) Methods, subtitle “Calculating the total human benefit of the intervention”, paragraph 1 and 2.  (*✓*) Methods, subtitle “Calculating the total livestock benefit of the intervention”, paragraph 1. |
| Statistical methods | 12 | (*✓*) Methods, subtitle “Analysing the total societal benefit of the intervention in relation to its total cost”, paragraph 1 and 2. |
| Results | | |
| Participants | 13 | (*✓*) Results, subtitle “Descriptive socioeconomic characteristics”, paragraph 1. |
|  |  |  |
| Descriptive data | 14 | (*✓*) Results, subtitle “Descriptive socioeconomic characteristics”, paragraph 1 and 2. |
| Outcome data | 15 | (*✓*) Results, subtitle “Project costs for the combined human and pig intervention”, paragraphs 1 to 10.  (*✓*) ResSubtitle “Pig intervention results” line 320. Table 5 |
| Main results | 16 | (*✓*) Results, subtitle “Extrapolated project cost”, paragraph 1  (*✓*) Results, subtitle “Determining the total cost-effectiveness of the intervention”, paragraph 1 and 2. |
| Other analyses | 17 | (*✓*) Sensitivity analysis, paragraph 1. |
| Discussion | | |
| Key results | 18 | (*✓*) Discussion, paragraph 1. |
| Limitations | 19 | (*✓*) Discussion, paragraph 5. |
| Interpretation | 20 | (*✓*) Discussion, paragraph 1. |
| Generalisability | 21 | (*✓*) Discussion, paragraph 3. |
| Other information | | |
| Funding | 22 | (*✓*) Described. |
